# Biocontrol of Diseases Caused by *Phytophthora capsici* and *P. parasitica* in Pepper Plants

**DOI:** 10.3390/jof9030360

**Published:** 2023-03-15

**Authors:** Mila Santos, Fernando Diánez, Brenda Sánchez-Montesinos, Victoria Huertas, Alejandro Moreno-Gavira, Belén Esteban García, José A. Garrido-Cárdenas, Francisco J. Gea

**Affiliations:** 1 Departamento de Agronomía, Escuela Superior de Ingeniería, Universidad de Almería, 04120 Almería, Spain; 2 Departamento de Agronomía, División Ciencias de la Vida, Campus Irapuato-Salamanca, Universidad de Guanajuato, Irapuato 36500, Guanajuato, Mexico; 3 Departamento de Biología y Geología, Edificio CITE IIB, Universidad de Almería, 04120 Almería, Spain; 4 Centro de Investigación, Experimentación y Servicios del Champiñón (CIES), Quintanar del Rey, 16220 Cuenca, Spain

**Keywords:** *Trichoderma*, *Paecilomyces*, biological control, root rot, blight, *Phytophthora*

## Abstract

The main objective of this study was to evaluate the ability of *Trichoderma aggressivum* f. *europaeum*, *T. longibrachiatum*, *Paecilomyces variotii*, and *T. saturnisporum* as biological control agents (BCAs) against diseases caused by *P. capsici* and *P. parasitica* in pepper. For this purpose, their antagonistic activities were evaluated both in vitro and in vivo. We analysed the expression patterns of five defence related genes, *CaBGLU*, *CaRGA1*, *CaBPR1*, *CaPTI1*, and *CaSAR8.2*, in leaves. All BCAs showed a high in vitro antagonistic activity, significantly reducing the mycelial growth of *P. capsici* and *P. parasitica*. The treatments with *T. aggressivum* f. *europaeum*, *T. longibrachiatum*, and *P. variotii* substantially reduced the severity of the disease caused by *P. capsici* by 54, 76, and 70%, respectively, and of the disease caused by *P. parasitica* by 66, 55, and 64%, respectively. *T. saturnisporum* had the lowest values of disease reduction. Reinoculation with the four BCAs increased the control of both plant pathogens. Markedly different expression patterns were observed in the genes *CaBGLU*, *CaRGA1*, and *CaSAR8.2*. Based on the results, all four BCAs under study could be used as a biological alternative to chemicals for the control of *P. capsici* and *P. parasitica* in pepper with a high success rate.

## 1. Introduction

The genus *Phytophthora* includes a group of devastating plant pathogenic species that economically affect important crops worldwide. This genus has long been included in the family *Pythiaceae*, within the group of oomycetes, but was ultimately included in the family *Peronosporaceae* after ribosomal analysis [1,2]. Advances in molecular analysis have enabled the elucidation of these issues and the description of new genera such as *Phytopythium* [3,4,5,6,7]. Currently, a total of 365 species and subspecies have been described (www.mycobank.org, accessed on 2 May 2022) in the genus *Phytophthora*, and this number continues to increase [5,8,9,10,11,12]. These species are classified into 12 phylogenetic clades [13,14,15], and new species of *Phytophthora* hybrids have been recently identified [16,17,18,19].

A number of species in this genus have been characterised as pathogenic in plants, which have a wide range of hosts. *P. capsici* Leonian and *P. parasitica* Dastur (syn. *P. nicotianae* Breda de Haan) are the most important pathogenic species of the genus for pepper (*Capsicum annuum*) crops in Spain [20,21]. *Phytophthora capsici* causes root rot, crown rot, foliar blight, and fruit rot in pepper [22]; *P. parasitica* is a causal agent of root and crown rot [20]. Due to the similarities of the symptoms on the roots and crown, both species may cause diagnostic confusion. The symptoms considerably vary according to the host, areas of infection, and environmental conditions, such as soil, air, and water temperature [23,24]. Methods of *Phytophthora* control include cultivation practices, fungicide application, and the use of resistant or tolerant varieties [25,26]. Control in many vegetable-growing areas in Spain has been based on the use of chemical soil disinfectants, many of which have been banned [27]. Currently, only a few fungicides are authorised for *Phytophthora* control, and their effectiveness is not guaranteed. In addition, they often generate resistance; for example, the resistance of *P. capsici* to metalaxyl [26,28,29]. Furthermore, the ability of *Phytophthora* to overcome the genetic resistance of plants owing to its genetic variability creates the need for alternative control methods for both diseases. Different cultivation techniques, such as grafting on resistant rootstocks [30,31,32,33,34] or nonchemical disinfection methods [35,36,37,38,39,40,41,42,43], have been used as alternatives. Crop rotation is a key component in the integrated management of diseases caused by *Phytophthora*; nevertheless, the survival of oospores, even in the absence of hosts, limits the effectiveness of these methods [21,44].

In recent years, different studies have been conducted by combining biological and chemical control agents (BCAs and CCAs, respectively) and/or combining the techniques mentioned above [45]. Reduced doses of fungicide stress and weaken the pathogen, increasing its susceptibility to attack by the antagonist [46]. BCAs are alternatives or complements to CCAs. CCAs are also adversely affected by the application of microbial antagonists because these antagonists are harmed by the application of pesticides, such that their effectiveness is sometimes weakened [47]. Biodisinfection and the subsequent incorporation of antagonistic bacteria and/or fungi may increase the benefits of this practice. For example, Wang et al. [48] demonstrated that combining biofumigation with the addition of *Bacillus amyloliquefaciens* controlled the disease caused by *P. capsici* by 40% to 90% in peppers. Other authors also described the benefits of the combined action of biofumigation and microbial incorporation [49].

Many microorganisms are growth inhibitors of *P. capsici* and *P. parasitica*, including *Streptomyces* spp. [50,51,52,53,54,55,56,57], *Bacillus* spp. [58,59,60,61,62,63,64,65], *Paenibacillus* spp. [66], *Pseudomonas* spp. [67,68], *Rhizobium* spp. [69], *Serratia* spp. [63,70], *Trichoderma* spp. [68,71,72,73,74,75,76,77], *Aspergillus* spp. [78,79], *Penicillium* spp. [80], *Curvularia* spp. [81], *Clitocybe nuda* [82], *Cladobotryum mycophilum* [83], *Fusarium solani* [68], *Aureobasidium pullulans* [84], *Rhodotorula mucilaginosa* [85], *Muscodor albus* [86], mycorrhizal fungi [87], and mixtures of microorganisms [88,89,90,91,92,93,94]. The mechanisms used for their control include the production of lytic enzymes, volatile and non-volatile active metabolites, mycoparasitism, competition for nutrients and space, and host resistance induction [75,95,96,97,98,99,100,101]. Similarly, soil bioactivation through the incorporation of microorganisms could reduce pathogen counts through the indirect effect of an optimised soil microbiome that improved the nonbiological factors of the soil [45]. Moreover, the rhizosphere microbiome plays a substantial role in reprogramming the defence responses of plants [102]. 

Plants recognise the presence of pathogens through interactions with receptors known as pathogen- and microbe-associated molecular patterns (PAMPs and MAMPs, respectively), inducing a local defence response termed PAMP-triggered immunity [103,104,105]. Some pathogens, including oomycetes, can suppress this response [105,106], which can be counteracted by cytoplasmic receptors (resistance proteins). These receptors, in turn, trigger a defence response termed effector-triggered immunity, which generates a hypersensitive response [105]. In addition to triggering local responses, plant pathogens induce systemic responses or systemic acquired resistance (SAR), such as *Fusarium oxysporum* fsp. *lycopersici* [107] and nonhost *Phytophthora nicotianae* [108], both of which protect pepper plants from subsequent infection with *P. capsici*. In addition to pathogens, numerous beneficial microorganisms trigger these immune responses [109,110]. Accordingly, systemic resistance against different *Phytophthora* species can be induced by *Trichoderma* [111,112,113,114,115], *Bacillus velezensis* [116], *B. subtilis* [115,117], *B. thuringiensis* [118], *B. vallismortis* [119], *B. amyloliquefaciens* [120], *Burkholderia* sp. [121], and the microorganisms present in aqueous compost extracts [122], among many others. The differential expression of genes involved in plant defence mechanisms allows us to compare how plants defend themselves against attack by different pathogens. Other control systems against *Phytophthora* include the inhibitory effect of extracellular self-DNA, which acts as a damage-associated molecular pattern (DAMP) on the pathogen, affecting the germination rate of *P. capsici* zoospores, thereby protecting the plant [123]. This type of technology should be studied in depth for subsequent applications in agriculture.

Pepper (*Capsicum annuum* L.) is the most important vegetable crop in Almeria, south-eastern Spain, covering 12,310 ha of cultivated land. In the 2020/2021 crop year, the total pepper production was 1,508,168 t, reflecting a 63.5% increase in the area of land cultivated with peppers in the last 10 years [124]. Biological pest control is performed on 96.3% of this cultivated land area. Biocontrol, using an antagonist, represents a potentially attractive disease management approach to reduce the side effects of fungicides as environmental pollutants. Therefore, the main objectives of this study were to determine (a) the potential of different BCAs against *P. capsici* and *P. parasitica* in vitro and in vivo; (b) the effect of volatile and non-volatile antifungal metabolites in vitro; (c) the effect of different BCAs on the development of diseases caused by both plant pathogens in vivo; and (d) the differential expression of the genes involved in plant defence responses, *CaBGLU*, *CaRGA1*, *CaBPR1*, *CaPTI1*, and *CaSAR8.2*, during the onset of marked symptoms in plants inoculated with both pathogens.

## 2. Materials and Methods

### 2.1. Fungal Isolates

**Figure 1:**
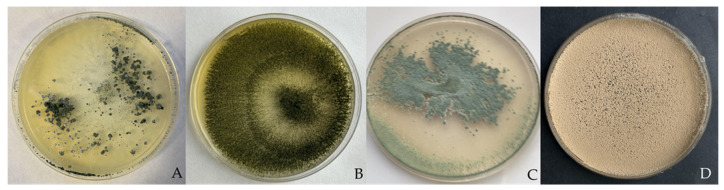
Fungal isolates used in this assay: (**A**) *T. saturnisporum*; (**B**) *T. longibrachiatum*; (**C**) *T. aggressivum* f. *europaeum*; (**D**) *P. variotii*.

The following BCAs were selected in this study: *Trichoderma aggressivum* f. *europaeum* Samuels & W. Gams (TA) [125,126], *T. longibrachiatum* Rifai (TL) [126], *Paecilomyces variotii* Bainier (PAE) [127], and *T. saturnisporum* Hammil (TS) [72]. TS has been previously described as a BCA for *P. parasitica* and *P. capsici* [72,126], and was used as a reference to compare the efficacy of the fungal isolates TA, TL, and PAE tested in this study (Figure 1). All isolates were deposited in the Phytopathology laboratory of the Department of Agronomy, Universidad de Almería, (UAL), Spain.

Plants infected with *P. parasitica* and *P. capsici* were collected from pepper crops in the province of Almeria (Spain). Stem sections with active lesions were cut and tissue pieces from the boundaries between healthy and discoloured areas were disinfected with 2% NaOCl for 2 min and then abundantly washed with sterile distilled water. These fragments were dried on sterile paper and subsequently placed on potato dextrose agar (PDA, Cultimed Panreac EU). Once the isolates were obtained, pathogenicity tests were carried out on pepper plants (*Capsicum annuum* L. cv. Acorde) using the methods of Diánez et al. [72].

BCA isolates were grown on PDA for 5 or 10 days at 25–27 ± 2 °C under dark conditions and were maintained on PDA at 4 °C. *Phytophthora parasitica* and *P. capsici* were maintained on V8 agar.

### 2.2. Dual Culture Bioassays

TA, TS, TL, and PAE were screened for their antagonism in vitro against *P. parasitica* and *P. capsici*. The antagonism assay was performed on PDA in Petri dishes using the dual culture method [128]. Plugs of 0.5 cm of mycelia of all fungi were cut from the growing edge of 10-day-old cultures with active growth of each isolate. The plugs were placed at the ends of Petri dishes with a distance of 8 cm between the two fungi and antagonist–phytopathogen. The antagonistic fungus was placed in the Petri dish 24 h before the pathogen. All plates were sealed with Parafilm^®^ and incubated in the dark at 25 °C until the controls reached the edge of the Petri dish. Radial fungal colony growth was measured daily. Results were transformed into percentages of mycelium growth inhibition (PIRM). These tests were carried out in quintuplicate. 

### 2.3. Antifungal Volatile and Non-Volatile Metabolite Bioassay

The antifungal activity of volatile organic compounds (VOCs) of TA, TS, TL, and PAE against *P. parasitica* and *P. capsici* was assessed using the procedure described by Phoka et al. [97] and bi-compartment dishes. All fungi were grown in PDA medium for 5 days at 25 °C in the dark. A 0.5-cm-wide plug of each antagonist fungus was placed 0.5 cm from the edge of the plate in a compartment. Similarly, in the other compartment of the plate, the pathogen was placed as described above, 24 h later. The plates were sealed with 3 layers of parafilm and incubated at 25 °C in the dark for 5 days. The fungal diameter was measured and compared with control plates (without antagonist). The experiment was performed in quintuplicate and repeated twice.

To determine the antifungal activity of non-volatile organic compounds (N-VOCs) of TA, TS, TL, and PAE, Erlenmeyer flasks (500 mL) containing 100 mL of PDB medium (Cultimed Panreac EU) were inoculated with two 0.5-cm-wide plugs of each antagonistic fungus. The flasks were incubated without stirring at 25 °C in the dark for 7, 14, 21, and 30 days. Mycelia were harvested by filtration through two layers of cheesecloth and the supernatant was filtered through sterile Millipore membranes (pore size 0.22 μm) and collected in sterile tubes. Filtrates were then incorporated and mixed with the cooled PDA at 5, 10, or 15% (*v*:*v*) and immediately poured into 50 mm Petri dishes [129]. The plates without filtrate served as control. A mycelial disc of 5 mm diameter of *P. parasitica* and *P. capsici* was put in the centre of the Petri plates. The cultures were incubated at 25 °C for 7 days. The colony diameter was measured and the percentage inhibition of the radial growth was calculated. Each assay was performed in quintuplicate.

### 2.4. Effects of TA, TS, TL, and PAE Isolates on the Severity of Phytophthora Blight in Pepper

TA, TS, TL, and PAE isolates were tested for biocontrol of *Phytophthora* blight (*P. capsici* and *P. parasitica*) in pepper plants (*Capsicum annuum* L., cv. Largo de Reus). The experiment was performed in two phases, one phase in a nursery and another in a greenhouse. Two independent experiments were conducted using completely randomised block designs.

**Figure 2:**
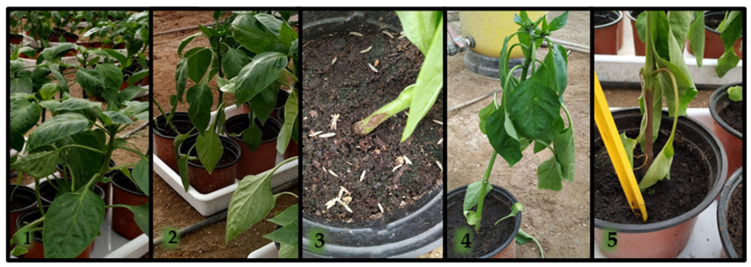
Disease severity scale for *Phytophthora capsici*; the same scale is used for *P. parasitica*. (**1**) Healthy plant; (**2**) symptoms beginning; (**3**) moderate symptoms; (**4**) severely affected plant; (**5**) dead plant.

Seedling was performed according to the procedure described by Sanchez-Montesinos et al. [130]. Pepper seeds were sown in 96-cell, commercial peat mix-filled, nursery polystyrene planting trays (70 mL volume) and covered with vermiculite. Trays were relocated to a greenhouse and rinsed with sterile distilled water (control) or a 5 mL (TA, TS, TL, or PAE) spore suspension per cell at 10^5^ spores per plant, after a 4-day period in a germination room (relative humidity (RH) = 95%; 25 °C). Two trays of seedlings for each treatment were cultivated under standard nursery culture conditions (18–28 °C; 75.4 ± 6.7% RH). After 45 days at the commercial nursery, 240 plants were transferred to pots (1 L capacity) containing peat moss, 40 plants of each antagonistic isolate, 40 control plants for each pathogen, and 40 plants for non-pathogen control. After transplanting, 50% of the plants were reinoculated with the same dose of the antagonist (R). After 7 days, all plants (except non-pathogen controls) were then inoculated with 5 mL of the zoospore suspension (10^4^ zoospores·mL^−1^) using a sterile micropipette, as described by Diánez et al. [72]. Symptom severity was rated periodically and final disease severity index was estimated according to the following scale [72]: 1, healthy plant; 2, symptoms beginning; 3, moderate symptoms; 4, severely affected plant; and 5, dead plant (Figure 2).

### 2.5. Effect of Antagonists on the Chlorophyll Content of Peppers

Chlorophyll content from the fourth leaf was determined using a SPAD 502 Plus Chlorophyll Meter (Konica Minolta, Inc., Ramsey, NJ, United States). The SPAD values were converted to chlorophyll using the formula described by Ling et al. [131]. The experiment was carried out in triplicates, with 10 plants measured at 15 and 45 days after transplanting (DATs).

### 2.6. RNA Extraction and Real-Time Polymerase Chain Reaction (RT-PCR)

The differential expression of the genes *CaBGLU* (*C. annuum* β-1,3-glucanase), *CaRGA1* (blight resistance protein), *CaBPR1* (basic PR protein 1), *CaPTI1* (ethylene responsive factor), and *CaSAR8.2* (Systemic Acquired Resistance 8.2) was determined using real-time PCR for all the treatments when the plants showed symptoms at stage 2 (62–65 days after the first application of the BCAs and 7–10 days after pathogen inoculation) and, similarly, in healthy plants inoculated with different BCAs (without pathogen). Gene expression was compared with the controls without inoculation. Leaves of a similar developmental stage were collected, frozen in liquid nitrogen, and kept at −80 °C until processing.

Total RNA was extracted from samples of pepper leaves using a commercial RNA PureLink RNA Mini Kit (Invitrogen), following the manufacturer’s manual. The samples were reduced to a smaller size and homogenised prior to the extraction with FastPrep-24 5G (MP Biomedical) for 40 s at a speed of 6 m/s. The quality and concentration of RNA was quantified by Nanodrop 2000 (Thermo Fisher Scientific, Waltham, MA, USA). In all cases, RNA concentrations were higher than 100 ng/µL and RNA extracts were stored at −20 °C. The high-capacity cDNA Reverse Transcription Kit (Applied Biosystems, by Thermo Fisher Scientific) was used to obtain cDNA from 1 μg of RNA. The cDNA was used as a template for the subsequent RT-PCR.

Quantitative RT-PCR was performed on a MyGo Pro^®^ RealTime PCR System using the SYBR Green fluorophore with the specific primers shown in Table 1. The SYBR Green reactions were performed in a 20 μL reaction mix comprising 1.5 ng of DNA, 10 μL of the SensiFAST SYBR No-ROX Kit (Bioline), and 2 μL of each of the primers (2 μM). The *ACT* gene was used as the housekeeping gene for data normalisation. In all reactions, amplifications were carried out under the following conditions: an initial hold step of 95 °C for 5 min and 45 PCR cycles of 95 °C for 15 s and 60 °C for 1 min. All *Ct* (cycle threshold) values were considered positive in the 18–35 range. Double delta *Ct* (ΔΔCT) analysis was used for determining relative expression [132] and the measurement of each gene was normalised with respect to the *ACT* gene. For each pair of primers, the melting curve was analysed to evaluate the specificity of the amplification, with high specificity in all cases. The visualisation of a single peak in the melting curve indicated a single specific fragment, the absence of primer dimers, and the lack of nonspecific products. For every experiment, mean values of six replicates are given for every concentration of samples tested, and their standard deviations are represented as error bars in figures.

### 2.7. Statistical Analysis 

The experimental results are presented as mean values (±standard deviation) for the different replicates. Mean separation was carried out using Fisher’s least significant difference (LSD) test. The data were tested by one-way analysis of variance (ANOVA) or Student’s *t*-test, with significance defined as *p*-values less than 0.05 (*p* < 0.05). Statgraphics Centurion 18 software was utilised for statistical analysis.

## 3. Results

### 3.1. Dual Culture Bioassays

**Figure 3:**
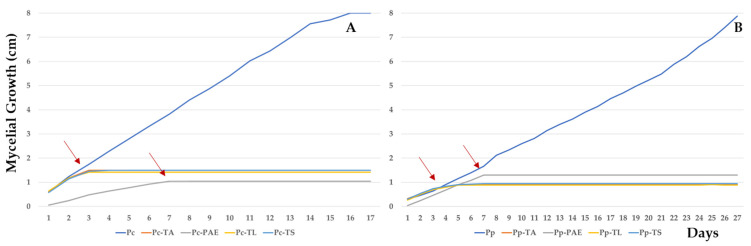
Mycelial growth of *P. capsici* (Pc) (**A**) and *P. parasitica* (Pp) (**B**) against *T. aggressivum* f. *europaeum* (TA), *P. variotii* (PAE), *T. longibrachiatum* (TL), and *T. saturnisporum* (TS), which peaked at 17 and 27 days, respectively.

All isolates showed high antagonistic activity against both *Phytophthora* species. *P. variotii* inhibition peaked at 83 and 87% for *P. parasitica* and *P. capsici*, respectively, at 7 days of incubation. The BCAs TA, TS, and TL showed similar high antagonistic activity values of approximately 88 and 82% for *P. parasitica* and *P. capsici*, respectively, and their activity peaked after 3 days of incubation (Figure 3).

### 3.2. Antifungal Volatile and Non-Volatile Metabolite Bioassay

**Figure 4:**
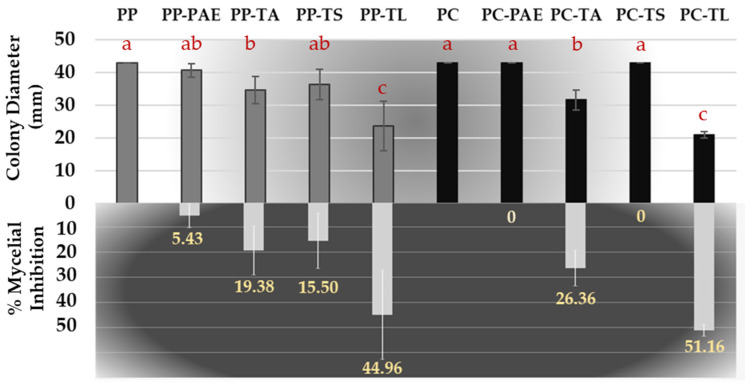
Colony diameter (mm) and mycelial inhibition (%) of *P. parasitica* (PP) and *P. capsici* (PC) by VOCs of *T. aggressivum* f. *europaeum* (TA), *P. variotii* (PAE), *T. longibrachiatum* (TL), and *T. saturnisporum* (TS). Mean values (±standard deviation) followed by different letters (line) indicate significant differences (*p* < 0.05) using the least significant difference (LSD) test.

The in vitro antifungal activity of VOCs (Figure 4) and N-VOCs (Table 2) produced by the isolates of TA, TS, TL, and PAE was tested against *P. parasitica* and *P. capsici*. The VOCs of TL and TA showed the highest percentages of growth inhibition for both plant pathogens of all isolates tested in this study, reaching approximately 50 and 20% inhibition, respectively. Conversely, the VOCs of PAE showed a weak growth inhibition effect against *P. parasitica* (5.43%) and no effect against *P. capsici*. Similarly, *P. capsici* growth was not affected by the VOCs of TS.

In turn, all N-VOCs showed a slight growth inhibition of both phytopathogens (Table 2). Unexpectedly, the N-VOCs presented an inhibition range of PC and PP lower than 20 and 15%, respectively, as well as lower PAE and higher TL growth inhibition values.

### 3.3. Effects of TA, TS, TL, and PAE Isolates on the Severity of Phytophthora Blight in Pepper

At the end of the assay, the plants not treated with the pathogens (T0) were asymptomatic. Plants inoculated with *P. capsici* and *P. parasitica* showed a mean disease rating of 5 and 4.2, respectively, with 100% incidence in both cases.

**Figure 5:**
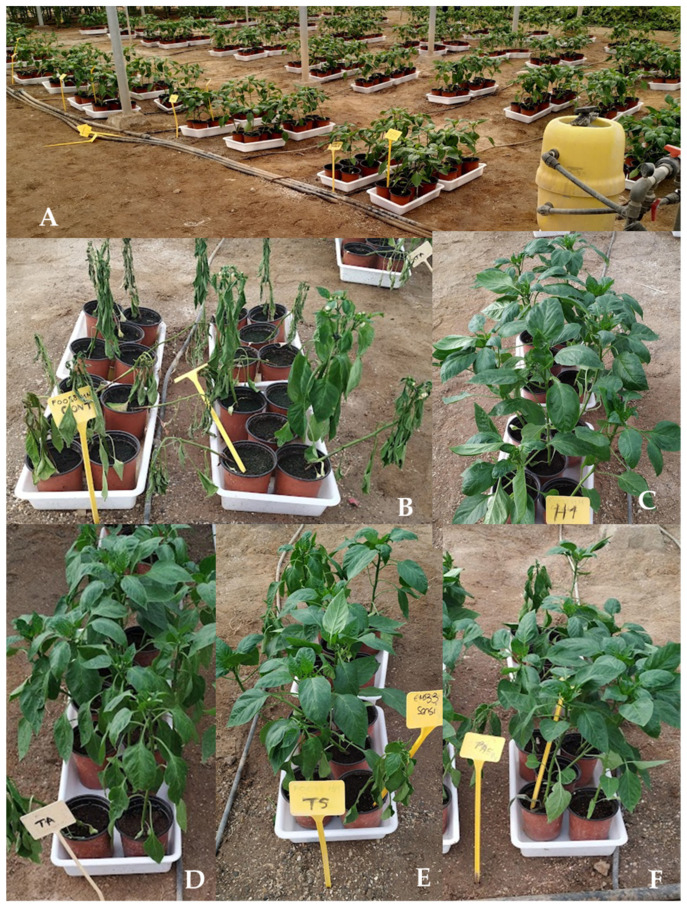
Effectiveness of BCAs in controlling disease caused by *Phytophthora* at end of assay (60 DATs): (**A**) distribution of plants in the greenhouse; (**B**) state of controls plants and plants treated with (**C**) *T. longibrachiatum* (TL), (**D**) *T. aggressivum* f. *europaeum* (TA), (**E**) *T. saturnisporum* (TS), and (**F**) *P. variotii* (PAE).

The in-plant antagonistic effectiveness of the BCA test strains against *P. parasitica* was higher than that against *P. capsici*. In both cases, nevertheless, the percentage of plants without symptoms was higher in plants reinoculated with the BCAs. No plants died when treated with PAE (considering a disease rating of 4 and 5), and plants treated with TL and with no symptoms reached a maximum disease rating of 2 (Figure 5).

Based on the results outlined in Table 3, the treatments with TL, PAE, and TA substantially reduced the severity of the disease caused by *P. capsici*, with 76, 70, and 54%, respectively, over untreated control plants (100% of mortality). Similarly, all reinoculation treatments provided better results, with TL showing the strongest antagonistic effect, reaching 78% disease severity reduction.

### 3.4. Effect of Antagonists on the Chlorophyll Content of Peppers

The chlorophyll content of plants infected with both pathogens and non-pathogens did not differ between treatments at 15 and 45 DATs. However, at 45 days, the plants inoculated with *P. capsici*, *P. parasitica*, and *Trichoderma longibrachiatum* showed a substantial increase in chlorophyll content, which reached 67 and 80% in both treatments (TL and TLR) for *P. capsici* and 56% for *P. parasitica* (TLR) in relation to the control (T0) (data not shown).

### 3.5. Molecular Responses of Pepper Leaves

**Figure 6:**
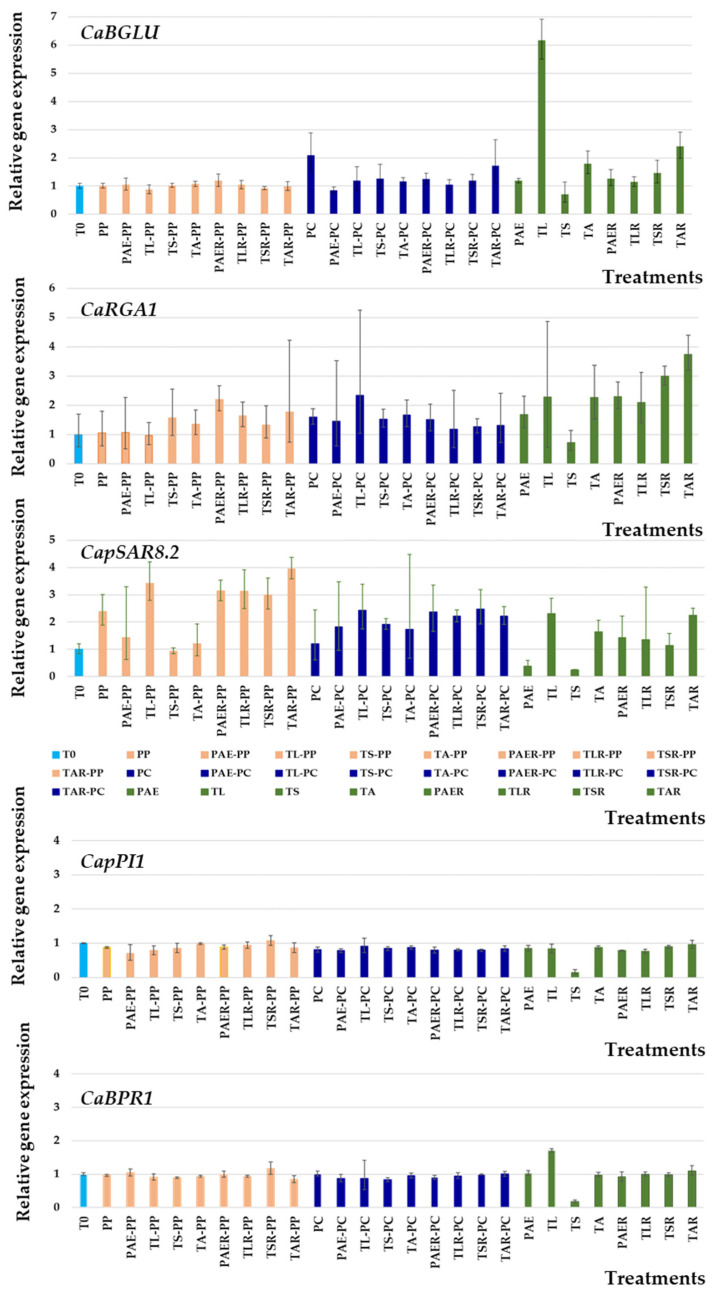
Analysis of the relative expression of defence-related genes *CaBGLU*, *CaRGA1*, *CaBPR1*, *CaPTI1*, and *CaSAR8.2*. Each bar represents relative gene expression for all conditions tested. Expressions of genes were normalised with respect to the *ACT* gene. Values were calculated following three replications, and standard deviations are shown.

Figure 6 shows the results from the analysis of the relative expression of the defence-related genes *CaBGLU*, *CaRGA1*, *CaBPR1*, *CaPTI1*, and *CaSAR8.2* at disease onset. This analysis was performed in leaves for all treatments with BCAs, comparing the results of these pepper plants with and without pathogen inoculation when they started showing symptoms (the samples were collected from plants with a disease severity rating of 2).

Considering the levels of each gene in relation to the levels of constitutively expressed *CaActin*, we observed that the expression levels of *CaBGLU* transcripts were not increased in plants treated with *P. parasitica* in any treatment tested in this study. However, inoculation with *P. capsici* produced the highest increase in the level of expression (two-fold), which decreased again after applying BCAs. Similarly, the *CaBGLU* gene was induced at low levels when applying BCAs only, except for TL, which increased the expression of this gene six-fold, and for TA and TAR, which doubled the expression of this gene.

The expression of the gene *CaRGA1* was moderately induced by BCAs, ranging from 1.68 to 4.69 times. These increased expression levels were maintained when incubating the plants with the pathogens. Strong induction of the *CaSAR8.2* gene also was found in pepper leaves treated with BCAs. For *P. parasitica*, the expression increased between 1.2 and four times, and the values were even higher upon reinoculation with BCAs. Expression was activated not only when inoculating with *P. capsici*, but when inoculating with BCAs. The application of BCAs alone showed a moderate level of expression activation in some cases, such as TL and TAR. The expression of the genes *CaBPR1* and *CaPI1* did not increase in any treatment.

## 4. Discussion

The importance of the biological activity of microorganisms close to plant roots has been highlighted in numerous studies on the biological control of oomycetes [137]. In soils rich in microorganisms, their competition for space and nutrients is intense, with a high production of numerous compounds and enzymes that limit the growth and development of plant pathogens, thus reducing the incidence of diseases. Moreover, many microorganisms stimulate plant growth or induce plant resistance to pathogens.

The control of diseases caused by oomycetes is particularly complex. Most of them produce effectors, which abolish or reduce plant defence responses against their attacks, and metabolites and enzymes, which degrade plant material, facilitating their penetration [138]. Numerous species of the genera *Trichoderma* and *Paecilomyces* have been reported to have fungicidal capacity against *Phytophthora* [72,139,140,141,142,143,144,145,146,147]. In the present study, the *P. capsici* and *P. parasitica* growth suppression effect of *T*. *aggressivum* f. *europaeum*, *T. longibrachiatum*, *T. saturnisporum*, and *P. variotii* was assessed in peppers.

In both in vitro and in vivo assays, the two plant pathogens showed differences in their relationship with BCAs. High antifungal activity (>80%) was observed in dual in vitro assays in PDA medium, with the plant pathogens reaching the maximum growth at 3 and 7 days of incubation for *Trichoderma* and *Paecilomyces* isolates, respectively. The three *Trichoderma* species completely overgrew the colony of the pathogen, showing hyperparasitism. Previous studies have shown the antifungal activity of these isolates against other plant pathogens, such as *Botrytis cinerea*, *Sclerotinia sclerotiorum*, and *Mycosphaerella melonis*, with a high efficacy [125,139]. These inhibition results are also very similar to those of Diánez et al. [72] when analysing *Trichoderma saturnisporum*. In turn, Ezziyyani et al. [146] found that *T. harzianum* provided inhibition values higher than 80% against *P. capsici*. Considering the variability of the protocols used in dual in vitro assays regarding the type and thickness of the solid culture medium in Petri dishes, the temperature, and the presence or absence of light, these results should be interpreted with caution because they are highly variable and often lack correlation between in vitro and in vivo conditions. Furthermore, the in vivo results depend on numerous factors, such as BCA dose, application time and method, and crop.

The *Trichoderma* species showed antifungal activity against *Phytophthora*. Nevertheless, the activity of N-VOCs and VOCs of *Paecilomyces* was very low or null. Volatile (VOCs) and non-volatile (N-VOCs) secondary metabolites of the *Trichoderma* and *Paecilomyces* species have different biological activities, such as biostimulation or biocontrol [147,148,149,150,151]. Li et al. [149] described 390 non-volatile components of 20 *Trichoderma* species, including *T. saturnisporum* and *T. longibrachiatum*, with antibacterial and antifungal capacity. In turn, Bae et al. [150] identified different non-volatile compounds produced by different *Trichoderma* species which showed the strongest inhibitory activities against *Phytophthora* isolates. Dai et al. [151] described 223 secondary metabolites and their biological activities isolated from different *Paecilomyces* species. Among them, only the compound farinomalein, isolated from *P. farinosus*, was a potent inhibitor of the plant pathogen *Phytophthora sojae*. Moreno et al. [152] did not assess high growth inhibition values of plant pathogens such as *F. solani* and *M. melonis* induced by N-VOCs and VOCs of *P. variotii*.

In our study, we found a high control of pepper plant diseases caused by *P. parasitica* and *P capsici*. The success of these results is derived mainly from inoculating BCAs in the seedbed phase. Consequently, when the plants were transplanted to the greenhouse, their roots were already colonised by BCAs and therefore “prepared” for a possible attack by phytopathogens, as clearly shown by the inability of reinoculation to significantly reduce disease severity, except for *T. saturnisporum*. In this case, reinoculation reduced disease severity by 57 and 70% for *P. parasitica* and *P. capsici*, respectively.

It has been reported that the addition of different species of *Trichoderma* in a plant’s rhizosphere induces resistance due to the rise in the amounts of defensive metabolites as well as enzymes, which act as elicitors [153]. In contrast to this assay, most studies aimed at identifying genes related to plant defence mechanisms against different pathogens are usually performed in the first hours after contact with elicitors, whether they are plant pathogens or beneficial microorganisms. We observed an increase in the relative expression of some plant defence-related genes, such as *CaBGLU*, *CaRGA1* and *CaSAR8.2*, when applying BCAs, except in the TS treatment. This exception could be directly related to the high disease expression shown despite the application of *T. saturnisporum*, which requires plant reinoculation for improved control. However, this hypothesis was not confirmed when applying BCAs together with both phytopathogens. Jung and Hwang [154] showed that the accumulation of *CaBGLU* mRNA on the stems of peppers infected with *P. capsici* was greatly reduced between 48 and 96 h, possibly due to deterioration of the infected stems. They concluded that pepper basic b-1,3-glucanase may mediate a part of the defence responses to pathogen infections. Conversely, the induction of defence-related genes, such as *CaPR1* and *CaBGLU*, is essential for SAR in pepper plants [155,156]. Additionally, some researchers have reported that the level and onset of β-glucanase expression is often positively correlated with the degree of resistance to the pathogen [156]. Accordingly, Jung and Hwang [154] observed that *CaBGLU* mRNA increased in the first stage of infection to similar levels in both compatible and incompatible interactions with *P. capsici*, but at later times, the gene had higher expression in the incompatible interaction. In our study, the expression of these genes was not increased in plants inoculated with *P. parasitica*, even though plants co-inoculated with BCAs showed some degree of resistance against disease and did not die. For *P. capsici*, the relative expression of the gene *CaBGLU* in leaves was low, but no correlation with a defence response was found since the maximum disease severity was reached in all control plants without BCAs, which showed a 2-fold expression induction.

A high number of disease resistance genes are induced by *P. capsici* invasion, such as *CaRGA1* [157]. The study of the expression of RGA genes under pathogen attack would facilitate the determination of whether they play an active role in resistance or if they are merely linked to resistance genes [157]. Our results showed a low induction of the expression of this gene in all treatments, which reached higher values when BCAs were applied without the pathogen, except for TS. The application of *P. parasitica* did not increase the relative expression of this gene.

Silvar et al. [158] observed a strong and rapid induction of the *CaBPR1* gene in an incompatible interaction of pepper plants with *P. capsici*. Similarly, overexpressing this gene in tobacco plants increased tolerance to *P. nicotianae* and to the bacterial pathogens *Ralstonia solanacearum* and *Pseudomonas syringae* pv. *tabaci* [159]. This gene apparently plays a key role in the ability of resistant pepper cultivars to restrict pathogen colonisation, which is conversely weak in susceptible genotypes. In our study, the expression of this gene did not increase under the conditions of this assay, corroborating the findings of Sarowar et al. [159]. The expression of the gene *CaPTI1* did not change either, in contrast to the results reported by Jin et al. [98], who highlighted the high expression levels of the gene *CaPTI1* after inoculation with *P. capsici*, which were higher in stems than in leaves.

The *SAR8.2* gene is a gene that controls plant resistance to *P. nicotianae* [160]. Lee and Hook [135] suggested that *CaSAR8.2* functions as a molecular marker gene for various biotic and abiotic stresses in pepper plants. The relative expression results may be directly related to the resistance response observed for both plant pathogens tested in this study, further highlighting that BCA reinoculation decreases disease incidence and severity, and in turn increases the expression of the *CaSAR8.2* gene.

The expression of these genes does not seem to be linked to the ability to develop symptoms, except for the gene *CaSAR8.2*. The results of these plants must be compared with those of plants without any symptoms and at times near inoculation as well. In our study, we were unable to clearly identify the genes involved in improving plant resistance. Genes involved in the early response of plants with resistant genotypes, such as *CaBPR1*, showed no changes in expression. However, the relative expression levels of genes involved in SAR responses were increased. In any event, the high variability of the results found in different samples or replicates makes it difficult to interpret the results. Therefore, further studies are needed to clarify the role that these genes play in reducing disease severity.

Biological control is presented as an ecological and healthy alternative to chemical control. As commented above, numerous studies have shown the different mechanisms of action that microorganisms use to control the growth and multiplication of the plant pathogens and pests that affect crops. This scientific development contrasts with the reality in the field. The preventive nature of this type of control, possible changes in crop management, and new pest and disease problems resulting from climate change, make it difficult to broadly implement such solutions. The withdrawal of numerous commonly used phytosanitary active ingredients has forced production systems to search for and develop new biological control agents. In addition, farmers and technicians must change their mindset for biological control to work. The use of BCAs from the seedbed, which reduces the inoculum levels of the pathogen in crops, combined with the use of plant varieties with some degree of resistance to some diseases and reduced doses of fungicide could provide high levels of disease control.

## 5. Conclusions

We are the first to describe *P. parasitica* and *P. capsici* control using *T*. *aggressivum* f. *europaeum* and *Paecilomyces variotii*. In addition, a marine isolate, *T. longibrachiatum*, showed a high capacity to suppress disease expression. BCA reinoculation increased plant survival and the percentage of plants without symptoms. Similarly, applying beneficial microorganisms moderately activated genes involved in the defence responses in pepper plants.

## Figures and Tables

**Table 1:** PCR primers used in SYBR Green assays.

Target	Primers	Sequences (5′→3′)	Reference
*CaBGLU*	CABGLU-F	ACAGGCACATCTTCACTTACC	[107]
	CABGLU-R	CGAGCAAAGGCGAATTTATCC	
*CaRGA1*	CARGA-F	ATGAGAAGGGAATAGGACGAG	[133]
	CARGA-R	ACATCCAATGGCAGGAAACT	
*CaBPR1*	CaBPR1-F	GTTGTGCTAGGGTTCGGTGT	[99]
	CaBPR1-R	CAAGCAATTATTTAAACGATCCA	
*CaPTI1*	CapPI1-F	TTTGAAACGGCCGAAGAAGC	[98,134]
	CapPI1-R	TGCACGATTCTGTCTTAGCGT	
*CaSAR8.2*	CapSAR8.2-F	TGTTGCCAGGGAGATGACTTC	[135]
	CapSAR8.2-R	ACAACGGCCATGACAAGTTT	
*ACT*	ACT-F	TGTTATGGTAGGGATGGGTC	[136]
	ACT-R	TTCTCTCTATTTGCCTTGGG	

**Table 2:** Mycelial growth of *P. parasitica* and *P. capsici* in PDA medium supplemented with 5, 10, and 15% microfiltered extract of TA, TL, TS, and PAE at different incubation times (7, 15, 21 and 30 days) in the dark and at 25 °C.

	Colony Diameter (cm)							
	*Phytophthora capsici*				*Phytophthora parasitica*			
	Incubation Time (days)							
Treatments	7	15	21	30	7	15	21	30
**Control**	2.7 ± 0 a*	2.68 ± 0.06 a	2.7 ± 0 a	2.7 ± 0 a	2.7 ± 0.00 a	2.69 ± 0.03 a	2.69 ± 0.08 a	2.7 ± 0.00 a
**PAE 5%**	2.65 ± 0.00 a	2.68 ± 0.06 a	2.66 ± 0.07 ab	2.68 ± 0.06 a	2.51 ± 0.25 cd	2.54 ± 0.22 bcd	2.54 ± 0.22 bcd	2.62 ± 0.11 b
**PAE 10%**	2.61 ± 0.10 ab	2.61 ± 0.18 ab	2.61 ± 0.18 abc	2.61 ± 0.18 bc	2.62 ± 0.09 b	2.62 ± 0.09 ab	2.60 ± 0.10 abc	2.59 ± 0.09 b
**PAE 15%**	2.66 ± 0.18 a	2.62 ± 0.15 ab	2.63 ± 0.15 abcd	2.63 ± 0.15 ab	2.58 ± 0.08 bc	2.57 ± 0.08 bc	2.52 ± 0.08 bcd	2.58 ± 0.08
**TA 5%**	2.5 ± 0.14 cd	2.43 ± 0.1 cd	2.53 ± 0.19 cde	2.60 ± 0.09 bc	2.58 ± 0.08 bc	2.45 ± 0.07 de	2.61 ± 0.04 ab	2.58 ± 0.05 bcd
**TA 10%**	2.51 ± 0.07 cd	2.46 ± 0.07 cd	2.62 ± 0.07 bcde	2.58 ± 0.06 bcd	2.51 ± 0.12 cd	2.43 ± 0.11 e	2.52 0.0 bcd	2.58 ± 0.06 bc
**TA 15%**	2.55 ± 0.09 bc	2.54 ± 0.05 bc	2.52 ± 0.04 de	2.56 ± 0.05 bcd	2.54 ± 0.05 bcd	2.56 ± 0.05 bcd	2.49 ± 0.06 cd	2.52 ± 0.06 bcd
**TS 5%**	2.54 ± 0.09 bc	2.51 ± 0.08 bcd	2.54 ± 0.16 cde	2.52 ± 0.07 d	2.7 ± 0.00 a	2.65 ± 0.07 ab	2.43 ± 0.06 d	2.48 ± 0.1 e
**TS 10%**	2.47 ± 0.07 cd	2.39 ± 0.16 de	2.45 ± 0.08 ef	2.43 ± 0.07 e	2.57 ± 0.05 bc	2.45 ± 0.13 de	2.46 ± 0.05 d	2.48 ± 0.06 e
**TS 15%**	2.34 ± 0.08 e	2.41 ± 0.09 de	2.48 ± 0.11 ef	2.43 ± 0.07 e	2.52 ± 0.07 cd	2.46 ± 0.19 cde	2.48 ± 0.11 d	2.45 ± 0.05 ef
**TL 5%**	2.45 ± 0.13 cd	2.58 ± 0.14 de	2.5 ± 0.12 de	2.55 ± 0.08 cd	2.57 ± 0.05 bc	2.54 ± 0.10 bcd	2.43 ± 0.06 d	2.51 ± 0.07 de
**TL 10%**	2.43 ± 0.08 de	2.31 ± 0.25 ef	2.46 ± 0.13 ef	2.43 ± 0.1 e	2.54 ± 0.05 bcd	2.28 ± 0.19 f	2.44 ± 0.09 d	2.48 ± 0.13 e
**TL 15%**	2.18 ± 0.13 f	2.22 ± 0.11 f	2.38 ± 0.14 f	2.43 ± 0.06 e	2.48 ± 0.08 d	2.42 ± 0.11 e	2.44 ± 0.1 d	2.4 ± 0.09 f
***p***	0.0000	0.0000	0.0000	0.0000	0.0000	0.0000	0.0000	0.0000

* Values in same column with different letters are significantly different according to one-way analysis of variance (ANOVA) followed by Tukey’s test at the 0.05 alpha level of confidence. Green: favourable; Orange: no effect compared to control.

**Table 3:** *Phytophthora parasitica* and *P. capsici* disease severity in pepper inoculated with TA, TS, TL, or PAE (10^5^ spores per plant) in two experiments in greenhouse conditions in which plants were inoculated with antagonist before the pathogen and before/after (reinoculated, R). All plants, except for controls (T0), were then inoculated with 5 mL of zoospore suspension (10^4^ zoospores·mL^−1^). Disease severity was assessed on a 1–5 scale, where 1 indicated free of infection (plants without symptoms) and 5 indicated dead plant.

	*P. parasitica*		*P. capsici*	
Treatment	Severity	Plants without Symptoms (%)	Severity	Plants without Symptoms (%)
**T0**	1.00 ± 0.00 c*	100%	1.00 ± 0.00 c	100%
**TI**	4.20 ± 1.13 a	0%	5.00 ± 0.00 a	0%
**TA**	1.40 ± 0.69 c	70%	2.30 ± 1.76 b	60%
**TS**	2.60 ± 2.06 b	60%	4.10 ± 1.37 a	5%
**TL**	1.80 ± 1.31 bc	70%	1.20 ± 0.42 c	80%
**PAE**	1.50 ± 1.08 c	80%	1.50 ± 0.84 bc	65%
**TAR**	1.20 ± 0.42 c	80%	1.50 ± 0.84 bc	75%
**TSR**	1.10 ± 0.31 c	90%	2.40 ± 1.57 b	50%
**TLR**	1.20 ± 0.42 c	80%	1.10 ± 0.31 c	90%
**PAER**	1.30 ± 0.67 c	80%	1.50 ± 1.26 bc	70%

* Values in same column with different letters are significantly different according to one-way analysis of variance (ANOVA) followed by Tukey’s test at the 0.05 alpha level of confidence.

## Data Availability

Not applicable.
